# Exploring the Abnormal Characteristics of the Ovaries During the Estrus Period of Kazakh Horses Based on Single-Cell Transcriptome Technology

**DOI:** 10.3390/biology14101351

**Published:** 2025-10-02

**Authors:** Wanlu Ren, Jun Zhou, Jianping Zhu, Jianguang Zhang, Xueguang Zhao, Xinkui Yao

**Affiliations:** 1 College of Animal Science, Xinjiang Agricultural University, Urumqi 830052, China; yaoxinkui@xjau.edu.cn; 2 Animal Husbandry Science and Technology Research and Extension Center of Tacheng Prefecture, Tacheng 834700, China; zhoujun19830728@163.com (J.Z.); 18095958045@163.com (J.Z.); 18197588887@163.com (J.Z.); 3 Animal Husbandry and Veterinary Station of Emin County, Emin 834600, China; xjemzxg@sohu.com

**Keywords:** scRNA-seq, Kazakh horse, ovary, cellular atlas, cell–cell communication

## Abstract

The ovary is among the earliest organs to undergo age-related degeneration, limiting the reproductive potential of elite horses and constraining the growth of the equine industry. Follicular development during estrus is a key determinant of fertility, yet the molecular mechanisms underlying its decline, particularly at the level of specific ovarian cell types, remain poorly understood in equids. Here, we constructed a single-cell transcriptomic atlas to investigate ovarian changes in Kazakh horses. Using single-cell RNA sequencing (scRNA-seq), we profiled 112,861 cells from follicle-containing and follicle-absent ovaries, identifying nine distinct ovarian cell types and their subtypes, each with distinct gene expression signatures. Functional enrichment analyses revealed cell type-specific engagement in biological pathways, including ECM–receptor interaction, PI3K-Akt signaling, and oxytocin signaling. Gene expression patterns indicated tightly regulated processes of ovarian activation and cell differentiation. Notably, stromal cells exhibited high expression of *ROBO2*, *LOC111770199*, and *TMTC2*, while smooth muscle cells (SMCs) were marked by elevated levels of *CCL5*, *KLRD1*, and *NKG7*. Moreover, cell–cell interaction analyses revealed robust signaling interactions among SMCs, endothelial cells, neurons, and proliferating (cycling) cells. Together, these findings provide a comprehensive single-cell transcriptomic map of normal and abnormal ovarian states during estrus in Kazakh horses, offering novel insights into the cellular mechanisms of follicular development and identifying potential diagnostic biomarkers and therapeutic targets for ovarian quiescence in equids.

## Simple Summary

The growth and development of the ovaries in horses is a highly complex biological process. During the development of the ovaries, some individuals may show abnormal development, which makes the breeding work for horses very challenging. Therefore, in order to explore this complex issue, we used single-cell transcriptome sequencing technology to analyze 10 samples. Through bioinformatics results, we identified nine cell types and subtypes. Through our research, we will provide the first complete single-cell transcriptome atlas of normal and abnormal horse oocytes, offering new insights into the cell heterogeneity, activation status, and gene expression programs related to follicular development.

## 1. Introduction

In mono-ovulatory species such as humans, horses, and cattle, follicular development occurs continuously throughout the female reproductive lifespan. Initially, follicles within each cohort grow at a similar rate until one (or occasionally two to three) becomes dominant. Horses, as seasonally polyestrous animals, exhibit estrus cycles from spring to early summer, with each cycle lasting approximately 21 days. Compared with bovine, ovine, and porcine ovaries, the equine ovary exhibits several unique anatomical features: the medulla is located externally, the cortex internally, and ovulation occurs exclusively at a specialized region called the ovulation fossa. Typically, only one oocyte is released per cycle, with multiple ovulations being rare. When a dominant follicle reaches a critical size, a deviation in growth rate occurs—dominant follicles continue to develop while subordinate follicles undergo atresia. Following ovulation, the ruptured follicle luteinizes to form a corpus luteum, which later regresses, thereby initiating the next estrous cycle [1].

Single-cell RNA sequencing (scRNA-seq) has revolutionized transcriptomic research by enabling gene expression analysis at single-cell resolution. Over the past decade, advances in cell isolation, automation, and sequencing protocols have dramatically improved the sensitivity, throughput, and cost-efficiency of scRNA-seq. Compared to bulk RNA sequencing, scRNA-seq offers significant advantages in resolving cellular heterogeneity, characterizing rare cell populations, and uncovering dynamic processes such as tissue development, immune response, and tumor progression [2]. These technological advances have been paralleled by the development of analytical methods [3,4] and comprehensive cell-type reference atlases across multiple species [5]. While scRNA-seq has been widely applied in murine models to explore developmental biology and disease mechanisms, its use in large animal species, including equids, remains limited.

The cellular and molecular mechanisms governing ovarian activation in equids are still poorly understood. This knowledge gap is due in part to several practical challenges: (1) The relatively small global equid population compared to livestock species such as pigs, cattle, and sheep, (2) The high economic value of horses, which makes the acquisition of ovarian tissue samples, particularly in numbers sufficient for scRNA-seq (*n* ≥ 3), technically and ethically challenging. To address these limitations, this study provides foundational single-cell transcriptomic data from the ovaries of Kazakh horses, offering new insights into equine ovarian biology. Importantly, the dynamics of equine follicular development and hormonal regulation closely mirror those of humans [1], making the horse a valuable comparative model. Given the ethical and technical constraints of studying germline specification and early gonadal development in humans [2], exploring ovarian biology in horses may shed light on a range of human reproductive disorders, including infertility, ovarian follicular dysgenesis, germ cell tumorigenesis, and other endocrine disorders.

In this study, we constructed a comprehensive single-cell transcriptomic atlas of ovarian tissues from Kazakh horses to characterize cellular heterogeneity and investigate the regulatory roles of distinct ovarian cell types. While recent studies have begun to uncover the pivotal roles of ovarian stromal cells in maintaining tissue homeostasis and contributing to disease [6,7,8], it remains unclear whether follicular cells, stromal cells, or both are primarily responsible for ovarian quiescence and dysfunction. Our findings aim to fill this gap by providing a high-resolution cellular framework that informs future research in equine and comparative reproductive medicine.

## 2. Materials and Methods

### 2.1. Experimental Animals and Study Location

All animal experiments were conducted between 21 February and 5 March 2024. A total of ten healthy adult Kazakh horses were selected and divided into two groups based on ovarian morphology:

DY group (dominant follicle present): horses exhibiting at least one ovarian follicle ≥ 2 cm in diameter, as confirmed by macroscopic examination (*n* = 5)

DB group (dominant follicle absent): horses with no macroscopically visible follicles on the ovaries (*n* = 5)

**Figure 1:**
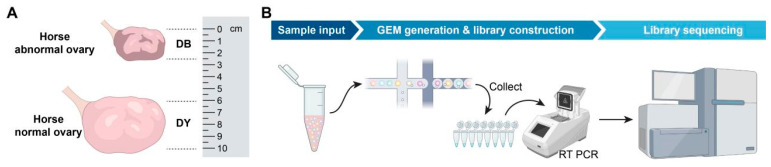
Sample collection and experimental workflow diagram. Notes: (**A**). Comparison chart of the sizes of horse abnormal ovary and horse normal ovary, (**B**). Single-cell sequencing flowchart.

Using B-type ultrasound diagnostic equipment, based on the development of the mare’s ovaries and follicles, it is determined that follicles with a diameter of at least 2 cm are dominant follicles. All the experimental horses were fasted for 12 h and underwent surgery the next day. This procedure is carried out by a licensed veterinarian, assisted by another practicing veterinarian and four assistants. After dissecting the abdominal wall, a piece of ovarian tissue was removed using a sterile scalpel and immediately stored in liquid nitrogen for future use. Antibiotic treatment was given to prevent infection. All the experimental horses were placed in Wantong Animal Husbandry Co., Ltd. in E’min County, Tacheng City, China, Xinjiang during the research period, and lived under uniform breeding conditions. All horses were provided with a standardized diet, fed high-quality dried alfalfa and crushed corn, and were free to drink water (Figure 1).

### 2.2. Experimental Workflow

#### 2.2.1. Preparation of Cell/Nucleus Suspensions

Fresh-frozen ovarian tissues were processed to obtain single-cell or single-nucleus suspensions by mechanical dissociation, followed by washing and resuspension in an appropriate buffer. Quality control criteria parameters included (1) intact nuclear membranes with no obvious damage; (2) nuclear concentration of 700–1200 nuclei/µL; (3) cell viability ≤5%; and (4) cell clumping rate <15%. Only suspensions that met all quality criteria were used for downstream capture using the 10× Genomics Chromium™ platform.

#### 2.2.2. Single-Cell/Nucleus Encapsulation Using the 10× Genomics Chromium™ Platform

The Chromium™ system uses microfluidic technology to partition individual cells or nuclei into nanoliter-scale oil droplets, together with gel beads coated with barcode oligonucleotides and lysis reagents. Upon lysis, released mRNAs hybridize to the poly(dT) regions of the barcoded primers. Reverse transcription is then carried out within each droplet, producing uniquely barcoded cDNA suitable for sequencing.

#### 2.2.3. Droplet Disruption g and cDNA Amplification

Following droplet breaking, barcoded cDNA was purified and enriched using magnetic bead-based clean-up. Amplification was performed via PCR, and the resulting cDNA was subjected to quality assessment before library construction.

#### 2.2.4. Library Construction

High-quality cDNA was enzymatically fragmented, end-repaired, and ligated with sequencing adapters and sample-specific indices to construct next-generation sequencing (NGS) libraries. Library integrity and concentration were assessed using standard quality control procedures prior to sequencing.

#### 2.2.5. Sequencing and Data Analysis

Sequencing was performed using the Illumina platform in paired-end 150 (PE150) mode, generating approximately 100 Gb of data (targeting~20,000 read pairs per cell). Raw data were processed using the Cell Ranger pipeline (10× Genomics) for initial quality control, read alignment, and generation of gene expression matrices based on the reference genome.

### 2.3. Single-Cell Subpopulation Classification Workflow

The single-cell transcriptomic data were analyzed using the Seurat package in R. The classification of cell subpopulations followed the standard Seurat pipeline, as outlined below:

1. Data normalization. Low-quality cells were excluded based on predefined filtering criteria. Gene expression levels were normalized using the LogNormalize method.
2. Dimensionality reduction. Principal component analysis (PCA) was conducted using the normalized expression values. The top 10 principal components (PCs) were then selected for subsequent clustering and subpopulation analysis.
3. Clustering and Subpopulation Identification. Graph-based clustering was performed using the following three key steps:
  a. K-nearest neighbor (KNN) graph construction: a KNN graph was constructed using Euclidean distances in the PCA space to represent pairwise similarities among cells.
  b. Edge weighting via Jaccard similarity: the edges in the KNN graph were weighted based on Jaccard similarity to refine intercellular relationships.
  c. Shared nearest neighbor (SNN) modularity optimization: cell clusters were identified using a modularity optimization algorithm on the SNN graph. This method enhances resolution and cluster stability by considering shared neighbors among cells.

Resulting clusters were annotated based on the expression of canonical marker genes, informed by the previous literature and publicly available cell atlases [6,9,10,11].

### 2.4. Quality Control

Post-QC metrics were evaluated to ensure data quality and suitability for downstream analyses. Violin plots were generated to visualize the distribution of key quality parameters, including the number of genes detected per cell, the total number of mRNA molecules, and the proportion of mitochondrial gene expression relative to total gene expression per cell (see Figure S1). The results confirmed high-quality sequencing output and appropriate complexity for single-cell analysis.

## 3. Results

### 3.1. Identification of Major Ovarian Cell Types in Kazakh Horses Using Single-Cell Transcriptomics

**Figure 2:**
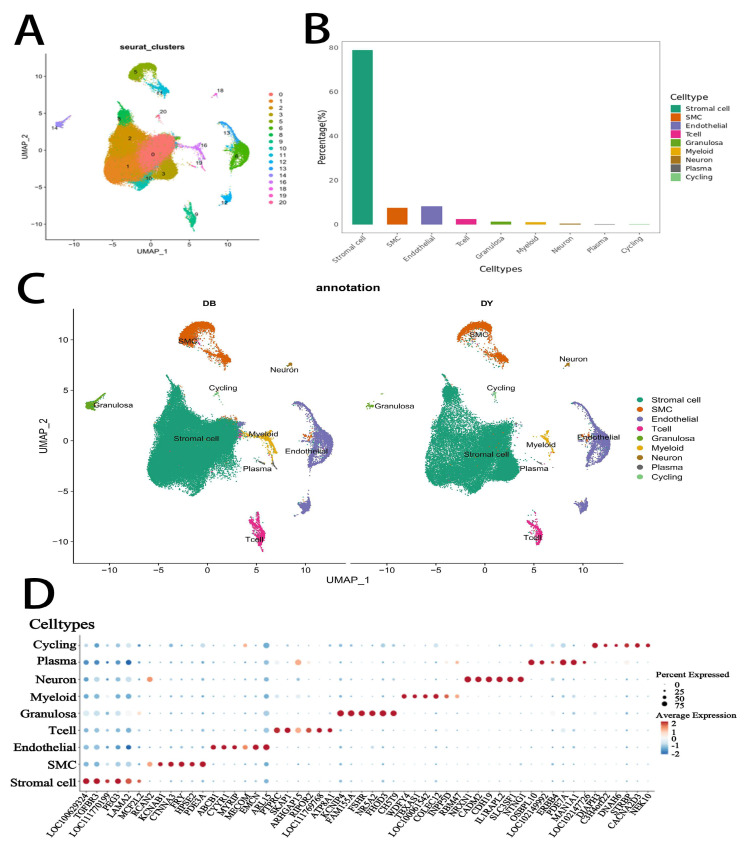
Identification of ovarian cell types in Kazakh horses by single-cell RNA sequencing. Notes: (**A**). UMAP of 21 cell clusters in Kazakh horse ovarian tissue, (**B**). Distribution of the 9 identified ovarian cell types, (**C**). UMAP showing 9 cell types across the two groups (DY and DB groups), (**D**). Bubble chart of major marker genes for the 9 ovarian cell types.

Single-cell RNA sequencing of ovarian tissues from Kazakh horses resulted in the identification of 21 transcriptionally distinct clusters (labeled 0 to 20; Figure 2A). These clusters were subsequently annotated into nine major distinct cell types based on canonical marker gene expression and transcriptomic signatures: stromal cells, smooth muscle cells (SMCs), endothelial cells, T cells, granulosa cells, myeloid cells, neurons, plasma cells, and cycling cells (clusters B and C). Among these, stromal cells represented the most abundant cell population, followed by SMCs and endothelial cells. The predominance of stromal and vascular-associated cell types highlights their potential importance in maintaining ovarian structure and function.

### 3.2. Cell Type-Specific Marker Gene Expression

To further characterize the identified cell populations, we analyzed the expression of representative marker genes across the nine identified cell types (2E). Stromal cells exhibited high expression of *LOC100629324*, *TGFBR3*, *PEG3*, and *LAMA2*, reflecting their role in extracellular matrix organization and tissue signaling. SMCs expressed genes such as *CTNNA3*, *FRY*, *HPSE2*, and *PDE3A*, consistent with their contractile and structural functions. Endothelial cells showed elevated levels of *ABCB1*, *CYYR1*, *EMCN*, and *ARL15*, supporting their vascular identity. Immune populations included T cells with strong expression of *PTPRC*, *SKAP1*, *RIPOR2*, and *LOC111769768*, and myeloid cells marked by *WDFY4*, *TBXAS1*, *and COLEC12*. Granulosa cells were characterized by high levels of *KCNIP4*, *FAM155A*, *FSHR*, *NR5A21*, *FHOD3*, and *CHST9*, indicative of their role in follicular development and hormone response. Neurons expressed *NRXN1*, *CADM2*, *CDH19*, *IL1RAPL2*, *SLC35F1*, and *NTNG11*, suggesting neuroendocrine regulation within the ovarian microenvironment. Plasma cells showed expression of *OSBPL10*, *ARHGAP153*, and *LOC100060608*, while proliferative (cycling) cells were defined by expression of cell cycle-related genes such as *TPX2*, *ECT2*, *CENPE*, and *CIT*. These marker gene profiles provide robust molecular evidence for the accurate classification of ovarian cell subtypes in Kazakh horses.

### 3.3. Differential Gene Expression Analysis Between Groups

**Figure 3:**
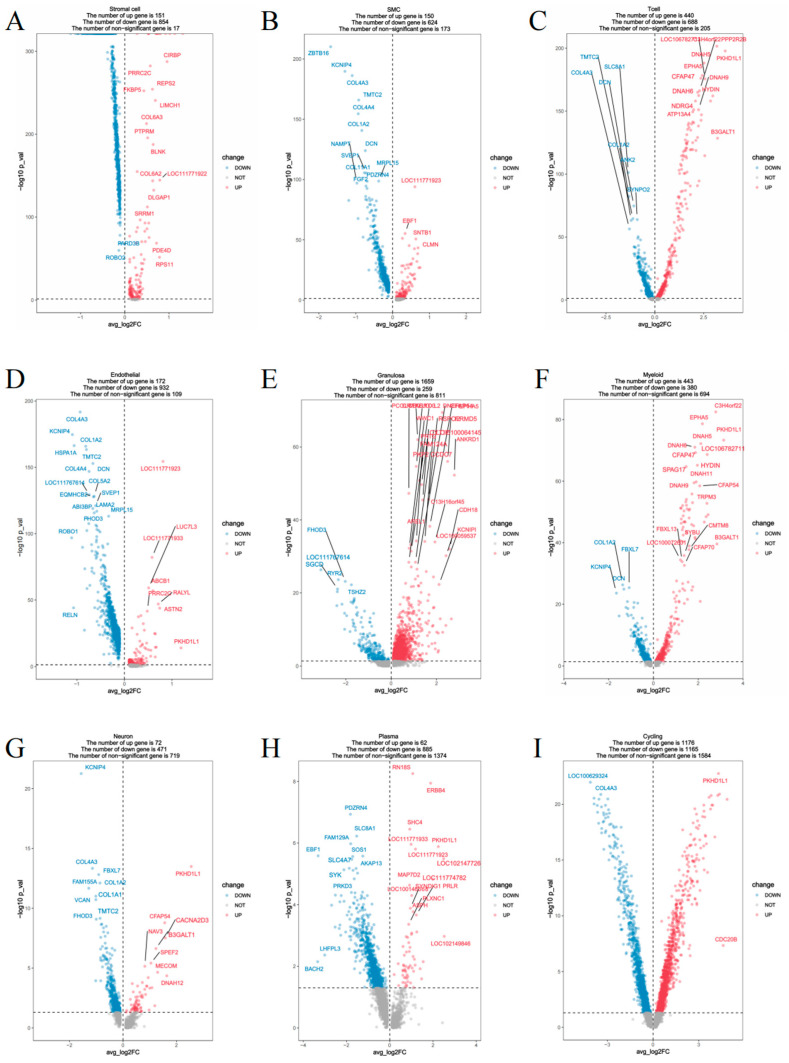
Volcano plots of significantly differentially expressed genes by cell type. Notes: (**A**). Stromal cells; (**B**). SMCs; (**C**). T cells; (**D**). Endothelial cells; (**E**). Granulosa cells; (**F**). Myeloid cells; (**G**). Neurons; (**H**). Plasma cells; (**I**). Cycling cells.

To elucidate transcriptomic differences associated with follicular status, differential gene expression (DGE) analysis was performed between the DY (follicle-present) and DB (follicle-absent) groups across all annotated cell types (Figure 3). Notably, substantial transcriptional changes were observed in multiple cell populations. In the comparison between the experimental group (DY group) and the control group (DB group), a large number of differentially expressed genes were identified. Circulating cells exhibited the most extensive transcriptional remodeling, with 1153 genes upregulated and 1169 genes downregulated. T cells also demonstrated considerable plasticity, with 402 genes upregulated and 289 genes downregulated. In contrast, granulosa cells showed a marked reduction in gene expression, with 1051 genes downregulated and only 245 genes upregulated. Other cell types, including bone marrow cells (307 upregulated, 315 downregulated), neuron cells (306 upregulated, 70 downregulated), endothelial cells (288 upregulated, 60 downregulated), and SMCs (224 upregulated, 85 downregulated), also exhibited transcriptional differences indicating functional shifts. Among stromal cells, 224 genes were upregulated and 85 downregulated, suggesting a dynamic role in supporting follicular activity. A few groups showed no significant differences in gene expression.

In addition, we identified 675 differentially expressed genes that lacked annotation, representing potential novel transcripts. These unannotated genes were distributed across cell types, with the highest numbers observed in cycling cells (*n* = 272), granulosa cells (*n* = 137), and plasma cells (*n* = 91), followed by myeloid cells (73), neurons (55), T cells (53), endothelial cells (43), SMCs (35), and stromal cells (34). These uncharacterized transcripts may contribute to species-specific ovarian biology and warrant further investigation.

### 3.4. GO and KEGG Enrichment Analyses

#### 3.4.1. Gene Ontology (GO) Enrichment Analysis of Upregulated Genes

To elucidate the functional implications of cell type-specific transcriptomic changes, we performed GO enrichment analysis of upregulated differentially expressed genes (DEGs) across major ovarian cell populations. Stromal cells showed enrichment primarily in translational machinery components, including cytosolic ribosome (CC), cytosolic small ribosomal subunit (CC), small ribosomal subunit (CC), and ribosomal subunit (CC), suggesting increased biosynthetic activity. SMCs were enriched in terms such as bone development (BP), cell leading edge (CC), and actin-based cell projection (CC), reflecting cytoskeletal dynamics and potential tissue remodeling roles.

In immune-related populations, T cells exhibited enrichment in cilium assembly (BP), embryonic pattern specification (BP), cilium organization (BP), and cilium (CC), indicating involvement in developmental signaling and intercellular communication. Myeloid cells showed specific enrichment in molecular function (MF) terms, including GTPase regulator activity, nucleoside-triphosphatase regulator activity, and guanyl-nucleotide exchange factor activity, implicating regulatory roles in intracellular signaling pathways.

Endothelial cells presented a broader enrichment profile (72 terms), including cell motility (BP) and neuronal cell body (CC), highlighting phenotypic plasticity and potential neurovascular interactions. Similarly, neuronal DEGs were enriched in 15 GO terms, prominently featuring neuronal cell body, consistent with neuroendocrine regulation within the ovary.

Granulosa cells exhibited the most extensive enrichment (93 terms), with top categories including mRNA metabolic process (BP), regulation of RNA splicing (BP), and regulation of organelle organization (BP), consistent with their high metabolic demand and endocrine functions. Plasma cells were enriched in associated processes such as cell junction assembly (BP), cell junction organization (BP), and mammary gland epithelium development (BP), pointing to developmental and epithelial differentiation signatures.

Cycling cells showed significant enrichment in processes associated with cell division and structural remodeling, including cilium organization (BP), cilium assembly (BP), and plasma membrane-bound cell projection assembly (BP), underscoring their proliferative and morphogenetic activity (full GO enrichment profiles are provided in Figure S2).

#### 3.4.2. Kyoto Encyclopedia of Genes and Genomes (KEGG) Enrichment Analysis of Upregulated Genes

KEGG pathway analysis was conducted to further explore the biological significance of upregulated genes across ovarian cell types. Stromal cell DEGs were significantly enriched in 208 pathways (*p* < 0.05), including ribosome, focal adhesion, and cytoskeleton in muscle cells. Similarly, SMC showed enrichment in 114 pathways, such as focal adhesion, cytoskeleton in muscle cells, and Vascular smooth muscle contraction, reflecting their contractile and structural properties.

T cell-associated DEGs were enriched in 236 pathways, prominently including long-term potentiation, oxytocin signaling pathway, and proteoglycans in cancer, suggesting involvement in signal transduction and immune modulation. Endothelial cells showed enrichment in 125 pathways, such as ribosome, coronavirus disease–COVID-19, and tight junction, highlighting their roles in barrier integrity and pathogen response.

Granulosa cells exhibited the broadest enrichment profile, with 309 significantly enriched pathways, including adherens junction, focal adhesion, and bacterial invasion of epithelial cells, underscoring their dynamic communication with the follicular microenvironment. Myeloid cell DEGs were enriched in 256 pathways, such as proteoglycans in cancer, parathyroid hormone synthesis, secretion and action, and phosphatidylinositol signaling system, indicating diverse regulatory and signaling functions.

Neurons were enriched in 86 pathways, including arrhythmogenic right ventricular cardiomyopathy, cytoskeleton in muscle cells, and purine metabolism, supporting a role in neuromodulatory and metabolic functions within the ovary. Plasma cell DEGs were associated with 117 pathways, such as ribosome biogenesis in eukaryotes, ribosome, and protein processing in the endoplasmic reticulum, reflecting their high biosynthetic activity. Cycling cell DEGs were enriched in 304 pathways, including ribosome, coronavirus disease–COVID-19, and motor proteins, consistent with active proliferation and protein synthesis (complete KEGG enrichment data are provided in Figure S3).

#### 3.4.3. GO Enrichment Analysis of Downregulated Genes

GO analysis of downregulated DEGs revealed enrichment in processes primarily associated with cytoskeletal organization and cellular metabolism. Stromal cells exhibited 194 significantly enriched GO terms, including actin cytoskeleton organization (BP), cytoskeleton organization (BP), and actin filament-based process (BP). SMCs and T cells shared similar enrichment profiles, with 49 and 119 GO terms, respectively, particularly enriched in cytoskeleton-related processes such as cell migration and actin filament organization (see Figure S4).

Granulosa cells showed enrichment in four metabolic terms, including glycosaminoglycan metabolic process (BP), aminoglycan metabolic process (BP), proteoglycan metabolic process (BP), and mucopolysaccharide metabolic process (BP). Myeloid cells had 178 enriched terms, including tube morphogenesis (BP), tube development (BP), and cell migration (BP). Plasma cells were enriched in 41 GO terms, including protein dephosphorylation (BP) and translation (BP). Cycling cells demonstrated 189 enriched terms, again dominated by cytoskeleton-associated categories, including actin cytoskeleton organization (BP), actin filament-based process (BP), and cytoskeleton organization (BP) (see Figure S5).

#### 3.4.4. KEGG Enrichment Analysis of Downregulated Genes

KEGG pathway analysis of downregulated DEGs revealed widespread suppression of signaling and cytoskeleton-related pathways across multiple cell types. Stromal cells showed significant enrichment in 275 pathways (*p* < 0.05), including focal adhesion, cytoskeleton in muscle cells, and regulation of actin cytoskeleton. Similar pathways were enriched in SMCs (257 pathways) and T cells (265 pathways), with consistent involvement of PI3K-Akt signaling and focal adhesion.

Endothelial cells exhibited enrichment in 281 pathways, including focal adhesion, cytoskeleton in muscle cells, and proteoglycans in cancer. In granulosa cells, 221 enriched pathways were identified, such as arrhythmogenic right ventricular cardiomyopathy, cytoskeleton in muscle cells, and axon guidance. Myeloid cell DEGs were enriched in 255 pathways, including focal adhesion, cytoskeleton in muscle cells, and ECM–receptor interaction, while neuron-associated DEGs were enriched in 251 pathways, including focal adhesion, cytoskeleton in muscle cells, and axon guidance.

Plasma cells displayed enrichment in 283 pathways, notably ubiquitin-mediated proteolysis, B cell receptor signaling pathway, and proteoglycans in cancer. Finally, cycling cells were enriched in 304 pathways, including cytoskeleton in muscle cells, focal adhesion, and ECM–receptor interaction (see Figure S6).

### 3.5. Cell Type Annotation and Stromal Cell Subclassification Between Groups

**Figure 4:**
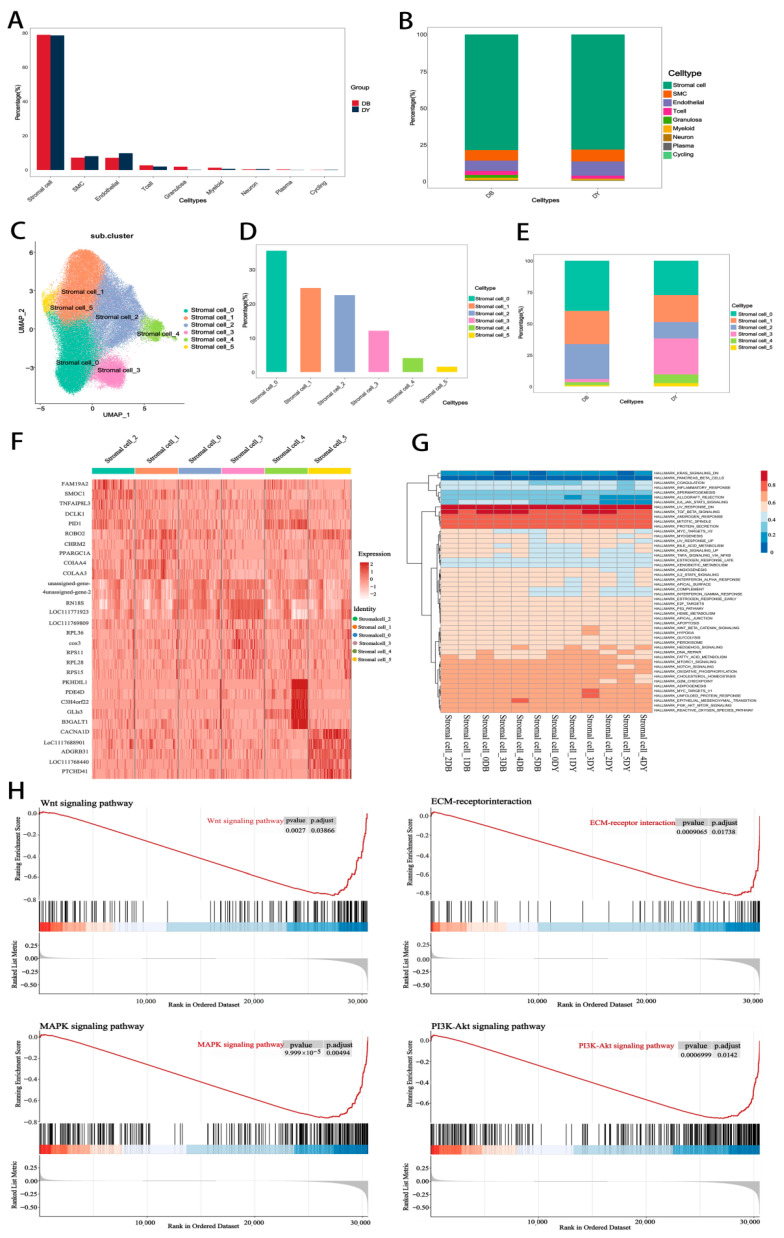
Expression Levels of Stromal Cell Subtypes. Notes: (**A**). Statistical chart of cell types in the DB group and DY group; (**B**). Proportion of cell type content within each group; (**C**). Principal component analysis diagram of cell subtypes; (**D**,**E**). Statistical chart of content of each cell subtype (**F**,**G**). Gene correlation heatmap within each cell subtype (**H**). GSEA Pathway Diagram.

As shown in Figure 4A,B, no qualitative differences in cell type composition were observed between the DB and DY groups. A total of nine cell types were consistently identified across both groups: stromal cells, SMCs, endothelial cells, T cells, granulosa cells, myeloid cells, neurons, plasma cells, and cycling cells. Among these, the five most abundant cell types were stromal cells, SMCs, endothelial cells, T cells, and granulosa cells, with stromal cells representing the most abundant in both groups.

Based on transcriptomic profiles, stromal cells were further subdivided into six subtypes (Figure 4C), designated as stromal cell 0 through stromal cell 5. In the DB group, stromal cells 0, 1, and 2 accounted for 40.02%, 26.30%, and 27.79% of stromal cells, respectively. In contrast, the DY group exhibited a redistribution of subtypes, with stromal cells 0, 1, 2, and 3 representing 27.37%, 21.40%, 13.11%, and 28.58%, respectively. Compared with the DB group, the DY group showed decreased proportions of stromal cells 0 and 2, while stromal cells 1, 3, 4, and 5 were expanded (Figure 4D–F). Notably, stromal cell subtype 4 exhibited specific expression of several marker genes such as *PKHD1L1*, *PDE4D*, *C3H4orf22*, *GLIS3*, and *B3GALT1* (Figure 4G), suggesting functional specialization. Gene Set Enrichment Analysis (GSEA) of stromal cell DEGs showed significant enrichment in pathways such as HALLMARK_UV_RESPONSE_DN, HALLMARK_TGF_BETA_SIGNALING, HALLMARK_ANDROGEN_RESPONSE, HALLMARK_MITOTIC_SPINDLE, and HALLMARK_PROTEIN_SECRETION (Figure 4H). These findings suggest that stromal cells are involved in hormone synthesis and metabolic regulation, providing both structural and functional support for follicular development.

### 3.6. Intercellular Communication and Pseudotime Trajectory Analysis of Ovarian Structural Cells

**Figure 5:**
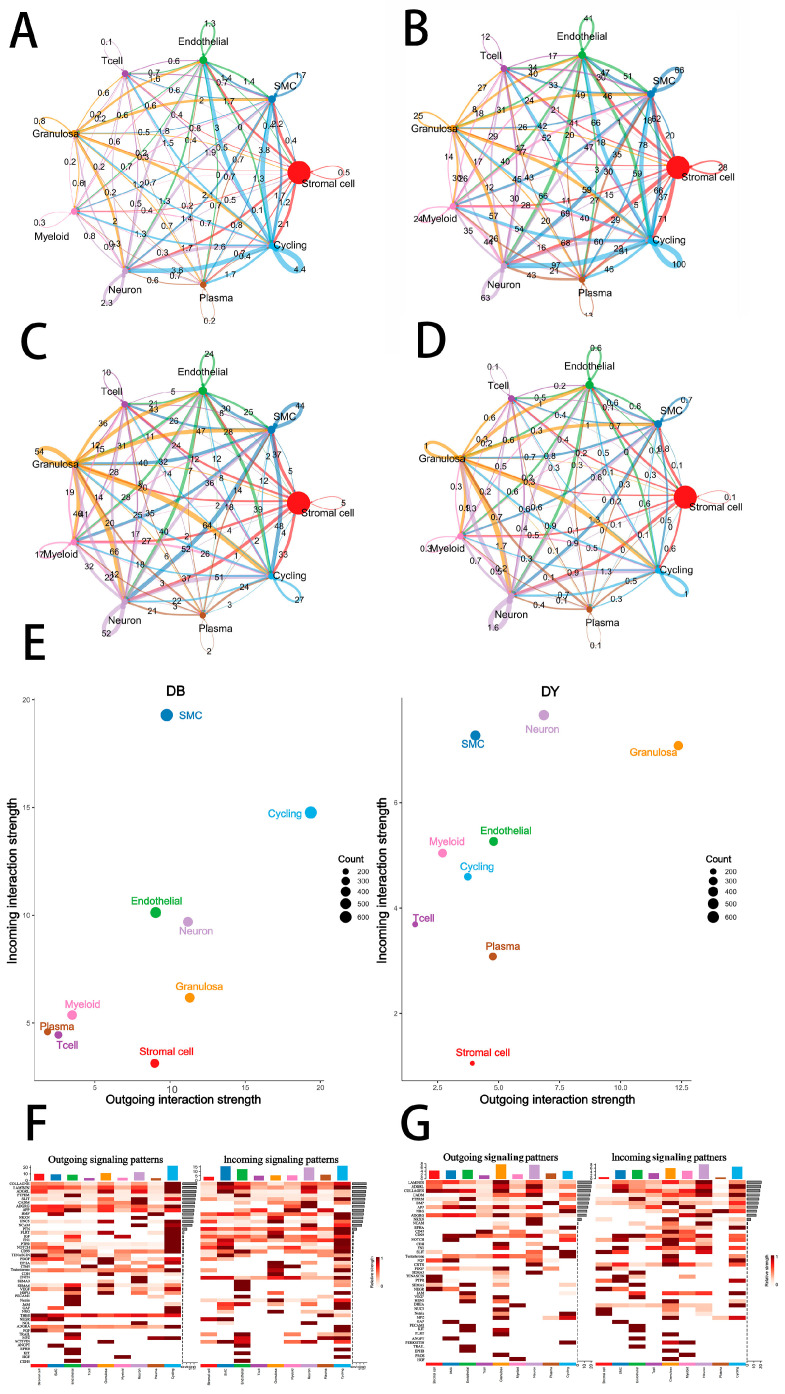
Cell–Cell Communication in Ovarian Tissue. Note: Colors represent different cell types. Circle size indicates the number of cells. Circles with outgoing arrows represent ligand-expressing cells; circles pointed to by arrows represent receptor-expressing cells. The thickness of the AC lines represents the number of ligand–receptor pairs between cell types, while the thickness of the BD lines indicates interaction strength between cell types. Panel (**A**–**D**) Cell Communication Diagram. Panel (**E**) shows scatter plots of incoming and outgoing interaction strength between cell types; Panels (**F**,**G**) show clustering analysis of cell–cell interactions in Kazakh horse ovarian tissue.

Cells in ovarian tissue engage in extensive intercellular interactions. The number and strength of signaling interactions in each group are shown in Figure 5A–D. In the DB group, SMCs were the primary signal-receiving cells, while cycling cells were the major signal-sending population. In contrast, the DY group displayed a different communication landscape, with neurons, granulosa cells, and SMCs receiving the most signals, and granulosa cells also serving as a major source of signaling output. These differences imply a remodeled intercellular communication network in the DY group, potentially linked to improved ovarian function and fertility potential. Notably, granulosa cells in the DY group exhibited stronger interactions with neurons, myeloid cells, cycling cells, and T cells, suggesting enhanced neuroendocrine and immune regulatory interactions compared with the DB group.

In the DY group, the granulosa cells exhibited significant enrichment of signaling ligands and pathways associated with cell adhesion, differentiation, and hormone regulation, including *LAMININ*, *BMP*, *NOTCH*, *PTN*, *FGF*, *NCAM*, *THBS*, *HSPG*, *CD45*, *CNTN*, *EPHB*, *DHEA*, *VISFATIN*, *ANGPTL*, and *CHOLESTEROL* (Figure 5F,G). Several of these, such as *LAMININ*, *NCAM*, and *CNTN*, are involved in maintaining extracellular matrix stability and cell adhesion, which support granulosa cell growth and follicular development. Pathways like *BMP*, *NOTCH*, *FGF*, and *EPHB* regulate granulosa cell proliferation, differentiation, apoptosis, and cell fate commitment—processes critical for follicular maturation and oocyte quality.

Granulosa cell function is further linked to hormonal synthesis and angiogenesis. Enrichment of *FGF*, *PTN*, *THBS*, and *ANGPTL* signaling in the DY group suggests improved intra-follicular angiogenesis, enhancing nutrient and oxygen supply. The DHEA and CHOLESTEROL pathways support steroidogenesis, with cholesterol serving as a precursor for steroid hormones and DHEA convertible to estrogen and androgens. These hormones are essential for follicle growth, ovulation, endometrial preparation, and pregnancy maintenance. The *CD45* pathway may play a role in local immune regulation within the ovary, maintaining tissue homeostasis and facilitating proper immune responses during fertilization and embryo implantation. Additionally, VISFATIN signaling may influence granulosa cell metabolism, signaling, and insulin sensitivity, supporting a stable endocrine environment necessary for reproduction.

Granulosa cells of the DY group also displayed enhanced outbound signaling via pathways such as *ADGRL*, *NOTCH*, *PTN*, *NETNN*, *TESTOSTERONE*, *RA*, *ANGPTL*, MIF, and TULP. These pathways are involved in cell maintenance and differentiation (PTN, NOTCH, ADGRL), reproductive microenvironment optimization (ANGPTL, MIF), hormonal regulation and signal transduction (TESTOSTERONE, RA), and potential neural and intracellular regulation (NETNN, TULP), further highlighting the functional sophistication of granulosa cells in the DY group.

**Figure 6:**
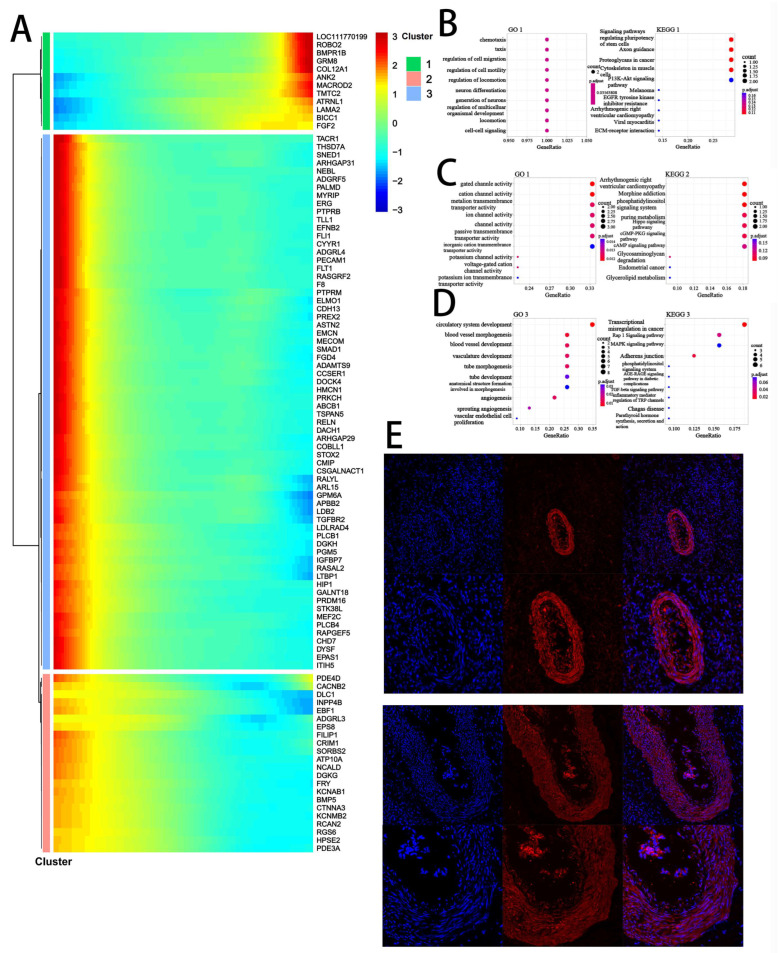
Pseudotime trajectory analysis of the temporal dynamics of stromal cells during ovarian aging. Notes: (**A**). Pseudotime heatmap illustrating the dynamic gene expression profiles associated with stromal cell fate commitment. Genes were grouped into four expression patterns using k-means clustering. The color gradient from red to blue represents high to low expression levels. (**B**–**D**). Top 10 enriched GO and KEGG terms for each gene cluster, showing dynamic gene functions based on stromal cells. (**E**). Immunostaining of Anti-Müllerian Hormone (AMH) in ovarian follicles. Immunohistochemical analysis was performed in both normal and abnormal ovaries. Scale bars: 200 µm and 500 µm.

To explore cellular differentiation trajectories, we performed pseudotime analysis on the structural cell populations, including stromal cells, SMCs, endothelial cells, and granulosa cells from DB and DY ovarian tissues. Pseudotime analysis, which arranges cells along a virtual developmental timeline based on gene expression similarity, revealed that endothelial cells and SMCs occupied earlier differentiation stages, whereas stromal cells and granulosa cells were positioned at later stages along two distinct developmental branches. Interestingly, one trajectory of stromal cells paralleled that of granulosa cells, suggesting a possible shared developmental fate. Cluster analysis based on pseudotime-associated gene expression identified three distinct gene clusters (Figure 6A). Cluster 1 genes (e.g., *LOC111770199*, *ROBO2*, and *GRM8)* were upregulated along the pseudotime axis and enriched in biological processes related to cell migration, neuron differentiation, and multicellular development. Cluster 2 genes (e.g., *TACR1*, *THSD7A*, and *SNED1)* exhibited a downward trend and were associated with gated and cation channel activity, as well as metal ion transport. Cluster 3 genes (e.g., *PDE4D*, *INPP4B*, and *EBF1*) shared a similar decreasing pattern and were enriched in angiogenesis-related terms such as circulatory system development and blood vessel morphogenesis. These results collectively suggest that stromal and granulosa cell differentiation in the DY group involve enhanced regulation of migration, metabolism, neural signaling, and vascular development—processes essential for follicular growth and reproductive competence.

### 3.7. Transcriptional Regulatory Landscape During Follicular Aging

**Figure 7:**
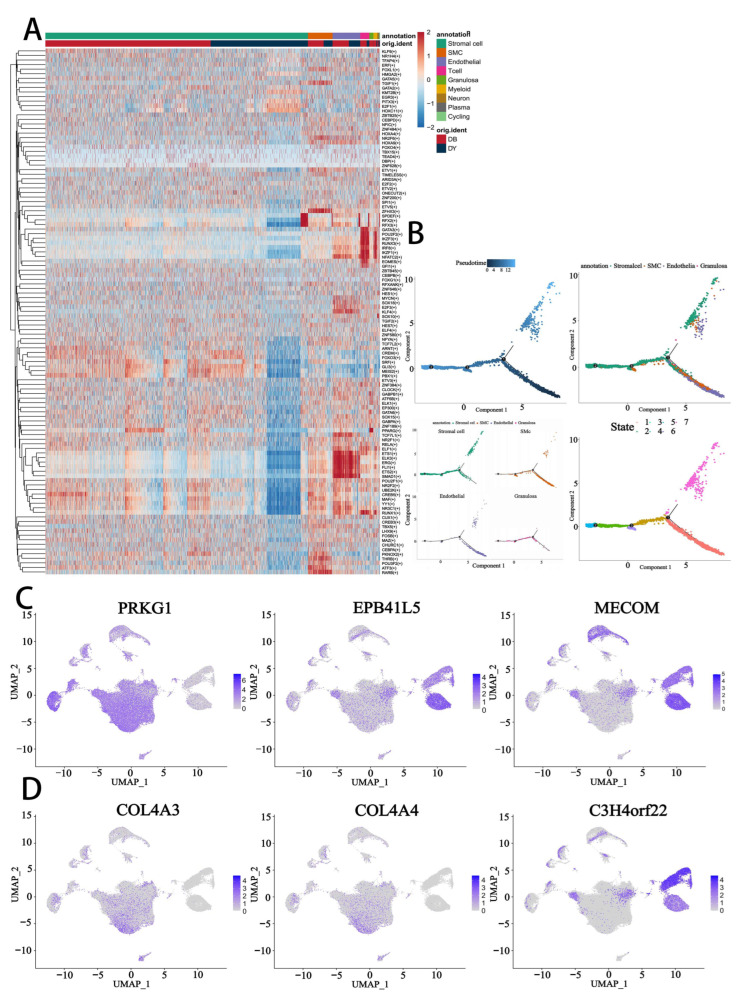
Transcriptional regulatory networks in normal and abnormal ovaries. (**A**). Heatmap showing key regulatory factors in ovarian cells identified by SCENIC analysis. The color gradient from red to blue indicates high to low regulatory scores. (**B**). Scatter plot illustrating divergent pseudotime trajectories for stromal cells, SMCs, endothelial cells, and granulosa cells in normal and abnormal ovaries. (**C**,**D**). t-SNE plots showing the expression of transcription factors in granulosa cells (GCs). The color gradient represents expression levels.

To further elucidate the regulatory mechanisms underlying follicular aging, we conducted transcription factor (TF) activity analysis across nine identified ovarian cell types from both DB and DY groups. Using SCENIC (Single-Cell Regulatory Network Inference and Clustering), we visualized the activity of TF regulons via heatmaps of AUC (Area Under the Curve) scores (Figure 7A), revealing cell type-specific transcriptional programs. Notably, several TFs exhibited dynamic regulation among cell types, suggesting their involvement in maintaining ovarian function or contributing to age-related decline.

Pseudotime trajectory reconstruction confirmed a developmental progression originating from endothelial cells and bifurcating into two main lineages leading to SMCs, stromal cells, and granulosa cells. Importantly, stromal and granulosa cells shared similar pseudotime trajectories, implying a possible convergence of differentiation fate (Figure 7B), consistent with our earlier findings of stromal–granulosa similarity.

Differential analysis of TF expression identified regulators such as *PRKG1*, *EPB41L5*, *MECOM*, *COL4A3*, *COL4A4*, and *C3H4orf22*, which displayed distinct cell type-specific expression patterns (Figure 7C,D). These TFs may serve as molecular markers for specific cellular identities or states during ovarian aging. Among them, *PRKG1*, *EPB41L5*, *CALD1*, and *FBXL7* emerged as key factors potentially associated with ovarian quiescence and structural maintenance (Figures S7 and S8). These findings suggest that age-related changes in TF activity may contribute to altered gene regulatory networks and reduced fertility potential in the aging ovary.

## 4. Discussion

In this study, we constructed the first single-nucleus transcriptomic atlas of equine ovarian tissue using 10× Genomics snRNA-seq technology, providing a comprehensive landscape of ovarian cell types and transcriptional programs in Kazakh horses. The ovary, as the primary reproductive organ in female animals, not only produces oocytes but also secretes sex hormones such as progesterone and estrogen, which are essential for reproductive function and secondary sexual characteristic development. Our analysis identified nine major cell types, among which stromal cells were the most abundant (41.3%), followed by follicular cells (26.1%) and granulosa cells (8.0%). Granulosa cells, though often discarded during assisted reproduction procedures, have been shown to possess oocyte reprogramming potential, offering promising applications in genetic conservation of valuable breeds.

This work represents the first single-cell analysis of equine ovarian and follicular development, complementing prior research in human, monkey, mouse, and bovine ovaries that identified granulosa cells, oocytes, stromal cells, and immune cells in adult ovaries [12,13,14,15]. In our study, we identified nine major cell types—stromal cells, smooth muscle cells (SMCs), endothelial cells, T cells, granulosa cells, myeloid cells, neurons, plasma cells, and cycling cells—as well as six stromal subtypes. Consistent with prior reports, specific marker genes such as *FIGLA*, *PRDM1*, *AMH*, *PDGFRA*, and *Cyp17a1A* were expressed in oocytes, germ cells, granulosa cells, stromal cells, and theca cells [16,17,18]. Interestingly, markers like *DAZL*, *DDX4*, and *OCT4*, which are commonly expressed in human ovaries, were not detected in goats, indicating interspecies differences in ovarian gene expression. Furthermore, we also identified a series of newly expressed protein-coding marker genes that are specific to different ovarian cell types, including *DAPL1, DRB, MYBPC*, *ECRG4*, *COL1A1*, *SOX18*, *KRT8*, *MYH11*, and *CTSW*, providing a valuable reference for ovarian cell identification in various livestock species.

In a study of human follicular fluid, Rosie M. Martinez and colleagues identified 11 signaling pathways; including extracellular matrix (ECM)–receptor interaction, focal adhesion, FoxO signaling, oocyte meiosis, PI3K-Akt signaling, adipocytokine signaling, AMPK signaling, cGMP-PKG signaling, ErbB signaling, gap junction, and GnRH signaling; which are associated with oocyte and follicular development [19]. Among these, ECM is crucial for ovarian development, folliculogenesis, and epithelial cell proliferation. The ECM provides structural stiffness to support dormant primordial follicles in the cortex [20,21,22]. When follicles are activated, they migrate to the medulla, which is mechanically softer than the cortex and more favorable for development [23]. ECM-related genes are also known to regulate ovarian reserve and follicle progression [24]. Studies in the bovine corpus luteum have shown that ECM–receptor interaction plays a key role in ovarian cellular function, consistent with our observations [25,26]. Moreover, ECM remodeling is essential for facilitating cell migration and angiogenesis during luteinization.

Through pseudotime analysis, we reconstructed the gene expression trajectories of different cell populations and clustered genes into three expression patterns. Among them, PRKG1, a cGMP-dependent protein kinase (PKG), was found to interact with SRC and participate in anti-apoptotic processes in high-grade serous epithelial ovarian cancer (HGS-EOC) [27]. ALD1, a cytoskeletal protein, has been linked with poor prognosis and platinum resistance in ovarian cancer [28]. Meanwhile, overexpression of *ESRP1* promotes mesenchymal-to-epithelial transition (MET), via alternative splicing of EPB41L5 and RAC1, suppressing migration while enhancing colonization [29]. The tumor suppressor *RNF180* and its downstream target *IPO4* have been validated in ovarian cancer models, forming a crucial molecular regulatory network [30]. A growing number of studies have emphasized the central role of the PI3K/AKT/mTOR pathway in the progression of ovarian cancer and chemotherapy resistance [31,32]. Our regression analysis identified six genes significantly associated with ovarian cancer: *PI3*, *TFAP2B*, *MUC7*, *PSMA2*, *PIK3C2G*, and *NME1* [33]. Moreover, *MECOM* has emerged as a promising therapeutic target in epithelial ovarian cancer. Initially annotated as two separate genes—*MDS1* and *EVI1*—*MECOM* is now recognized as a unified transcriptional unit with splice variants contributing to its complexity [34].

In addition to cancer-related findings, genetic studies in livestock have uncovered candidate genes associated with reproductive traits. Abdul Sammad identified *CACNB2*, *SLC39A12*, and *ZEB1* through a genome-wide association study (GWAS). Several single-nucleotide polymorphisms (SNPs) in *CACNB2*, *SLC39A12*, and *ZEB1* were significantly associated with reproductive traits, such as g.33258186G/A and g.33267172C/T in *CACNB2*, g.32751518G/A in *SLC39A12*, and g.34066997C/G and g.34063562C/G in *ZEB1*. A novel SNP g.33258042G/T was discovered in an intron of *CACNB2*. Furthermore, expression levels of these genes in granulosa cells varied significantly across different stages of follicular development [35].

This study identified 675 previously unannotated genes in equine ovarian tissue. This finding provides an important new perspective for enriching the genomic landscape of the horse ovary and opens new avenues for advancing our understanding of the biomolecular basis of equine reproduction. Genome annotation completeness is an essential foundation for elucidating tissue-specific functions; however, current horse genome annotations remain incomplete, particularly in reproductive tissues. These tissues often harbor numerous temporally and spatially specific genes whose functions are still underexplored. The identification of these unannotated genes not only addresses gaps in equine ovarian genome annotation but also indicates the potential existence of unique gene regulatory networks within the ovary. Moreover, these genes may play key roles in fundamental physiological processes such as ovarian development, folliculogenesis, and steroidogenesis.

Preliminary analysis of gene characteristics revealed that approximately 38% of the unannotated genes contain conserved sequences, as determined by homology comparisons with public databases (e.g., Ensembl, NCBI RefSeq) and other species (e.g., human, mouse, bovine). Among these, 12% harbor domains associated with germ cell development (e.g., zinc finger domains, RNA-binding domains), suggesting potential roles in regulating gene expression and cell fate determination. In addition, 27% of the genes, although lacking clear homologous sequences, were validated through transcriptome data as being highly and specifically expressed in equine ovarian granulosa cells or oocytes, further supporting their tissue-specific functional relevance [36]. GO functional enrichment analysis further demonstrated significant enrichment in biological processes such as follicle-stimulating hormone response, regulation of oocyte maturation, and G2/M phase transition of the cell cycle. These findings closely reflect the developmental dynamics of equine ovarian follicles from primordial to mature stages and provide a novel set of candidate genes for elucidating the molecular mechanisms underlying follicular development.

Current challenges in equine reproduction, such as ovulation disorders and low embryo implantation rates, remain without well-defined molecular markers or regulatory targets. Among the unannotated genes identified in this study, several displayed significant differential expressions between ovarian tissues of high-ovulation-rate breeds (e.g., Arabian horses) and low-ovulation-rate breeds (e.g., Heavy Draft horses). Notably, *LOC111770199* showed a positive correlation with follicle count, suggesting its potential as a molecular marker for evaluating equine reproductive performance [37,38]. Furthermore, cross-species comparison with unannotated ovarian genes from other livestock (e.g., cattle, pigs) revealed that only 23% exhibited conservation. This limited overlap may reflect the evolution of unique reproductive strategies in equids and provides valuable insight for exploring species-specific reproductive regulatory mechanisms.

## 5. Conclusions

This study provides the first comprehensive single-cell transcriptomic map of normal and abnormal equine ovaries, offering novel insights into cellular heterogeneity, activation status, and gene expression programs associated with follicular development. By delineating the developmental trajectories and transcriptional features of ovarian cell subpopulations, we uncovered key molecular mechanisms driving follicular dynamics in horses and established a framework for evaluating the reproductive potential of Kazakh horses. Moreover, at the single-cell level, we identified several critical genes and signaling pathways, including FST, SOX4, HIF1A, Wnt, and Myc target pathways, that may serve as promising targets for improving ovarian function in equids. Furthermore, the identification of 675 previously unannotated genes enriches the genomic landscape of the equine ovary and provides valuable resources for future studies on fertility regulation, ovarian biology, and breeding in livestock species. Together, these findings establish a foundational resource for understanding equine ovarian biology at single-cell resolution and provide important insights for reproductive trait improvement, ovarian function regulation, and the identification of molecular targets in fertility-related research and breeding programs. Due to the absence of functional validation experiments in this study, our team is currently focusing on primary cell cultures of different cell subtypes, with the aim of conducting further in vitro investigations.

## Data Availability

The datasets from this study are available in online repositories. The specific repository/repositories and their accession number are provided below: NCBI BioProject accession number: PRJNA1250130.

## Supplementary Materials

The following supporting information can be downloaded at: https://www.mdpi.com/article/10.3390/biology14101351/s1, Figure S1: Relative expression chart of each gene; Figures S2 and S3: GO annotation terms and KEGG signaling pathways upregulated in each cell subtype; Figures S4–S6: GO annotation terms and KEGG signaling pathways downregulated in each cell subtype; Figure S7: Relative gene expression map in each cell subtype; Figure S8: Relative expression levels of cell subtypes across groups.
