# Supplementary figures and images for: Exploring the Abnormal Characteristics of the Ovaries During the Estrus Period of Kazakh Horses Based on Single-Cell Transcriptome Technology
Source: Biology (Basel). 2025 Oct 2;14(10):1351. doi: 10.3390/biology14101351 (PMC12562243; doi:10.3390/biology14101351)

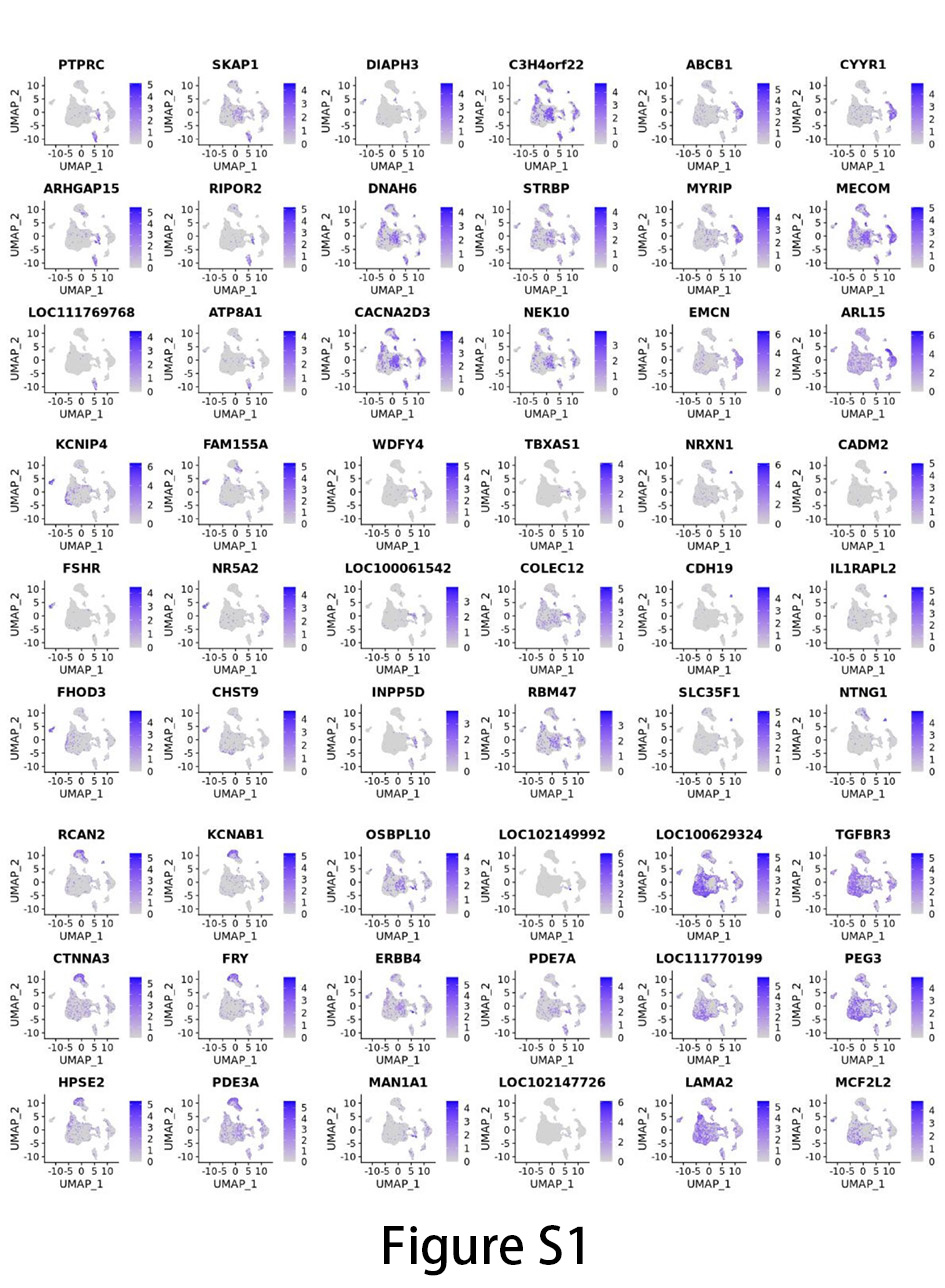

Supplement: Supplementary file 1 [file biology-14-01351-s001.zip › Figure S1.jpg]

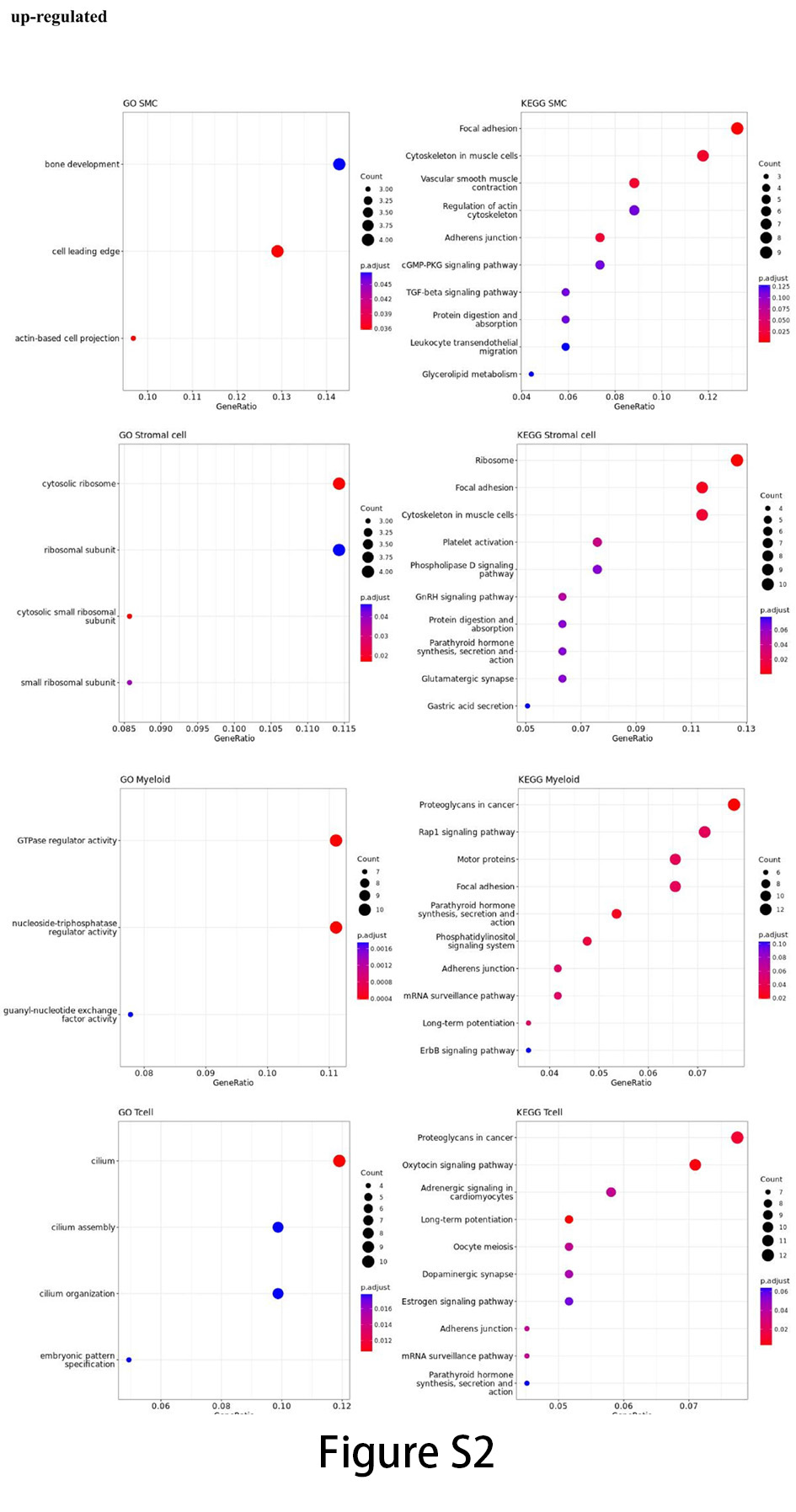

Supplement: Supplementary file 1 [file biology-14-01351-s001.zip › Figure S2.jpg]

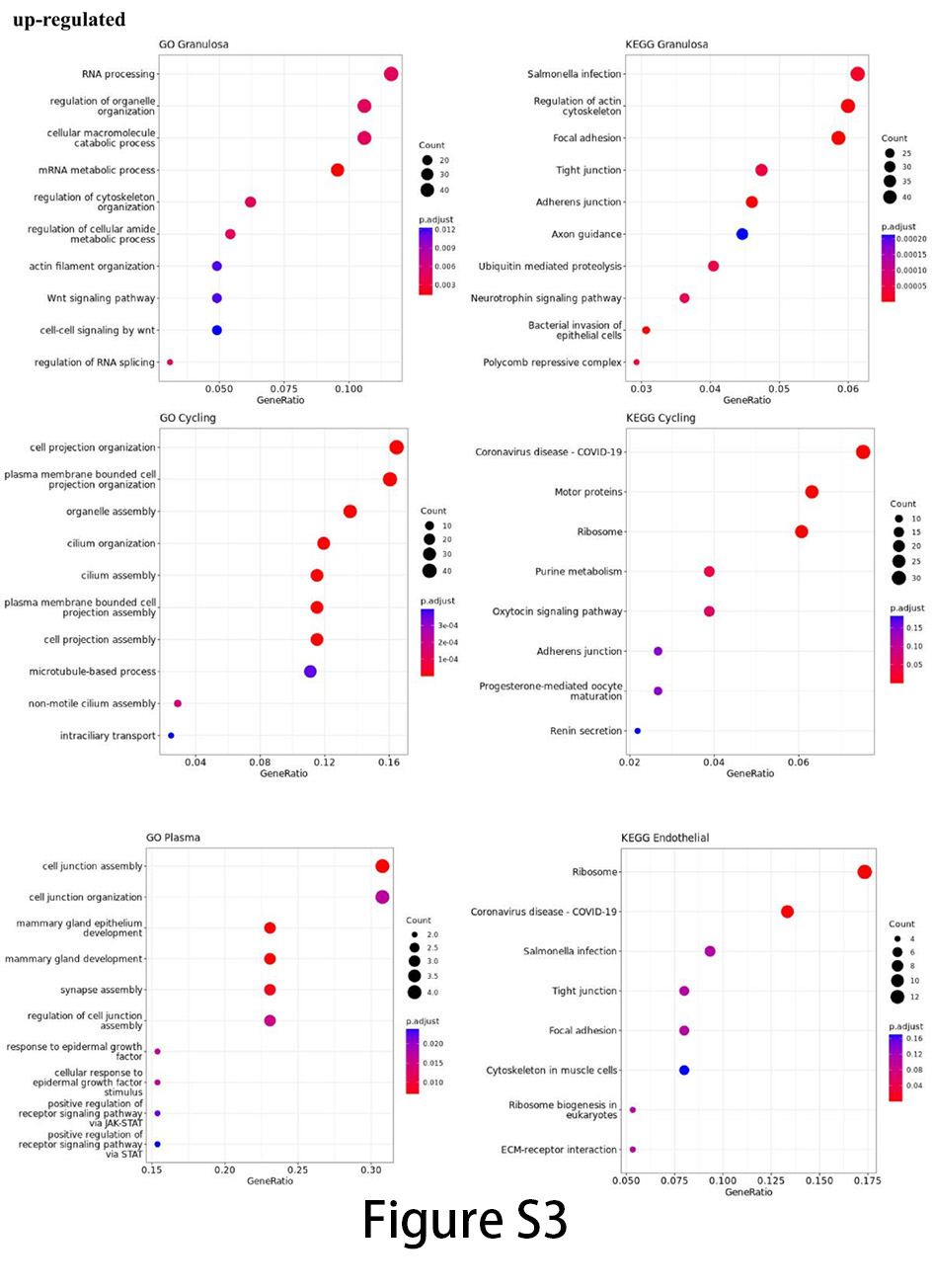

Supplement: Supplementary file 1 [file biology-14-01351-s001.zip › Figure S3.jpg]

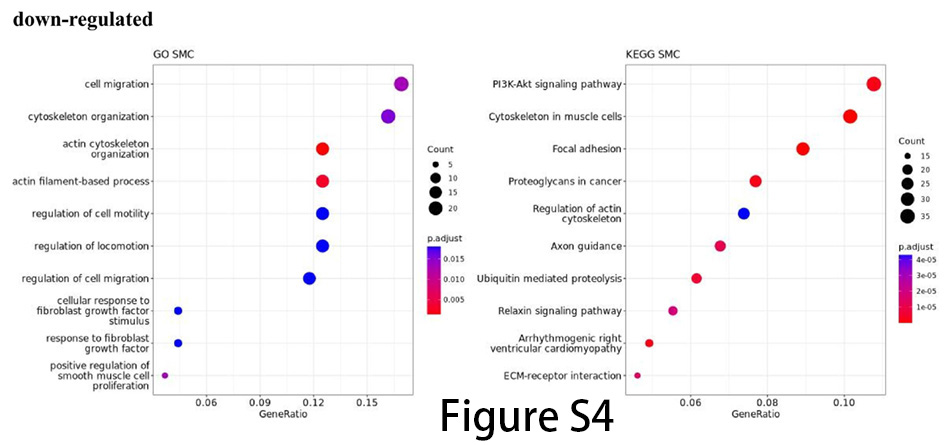

Supplement: Supplementary file 1 [file biology-14-01351-s001.zip › Figure S4.jpg]

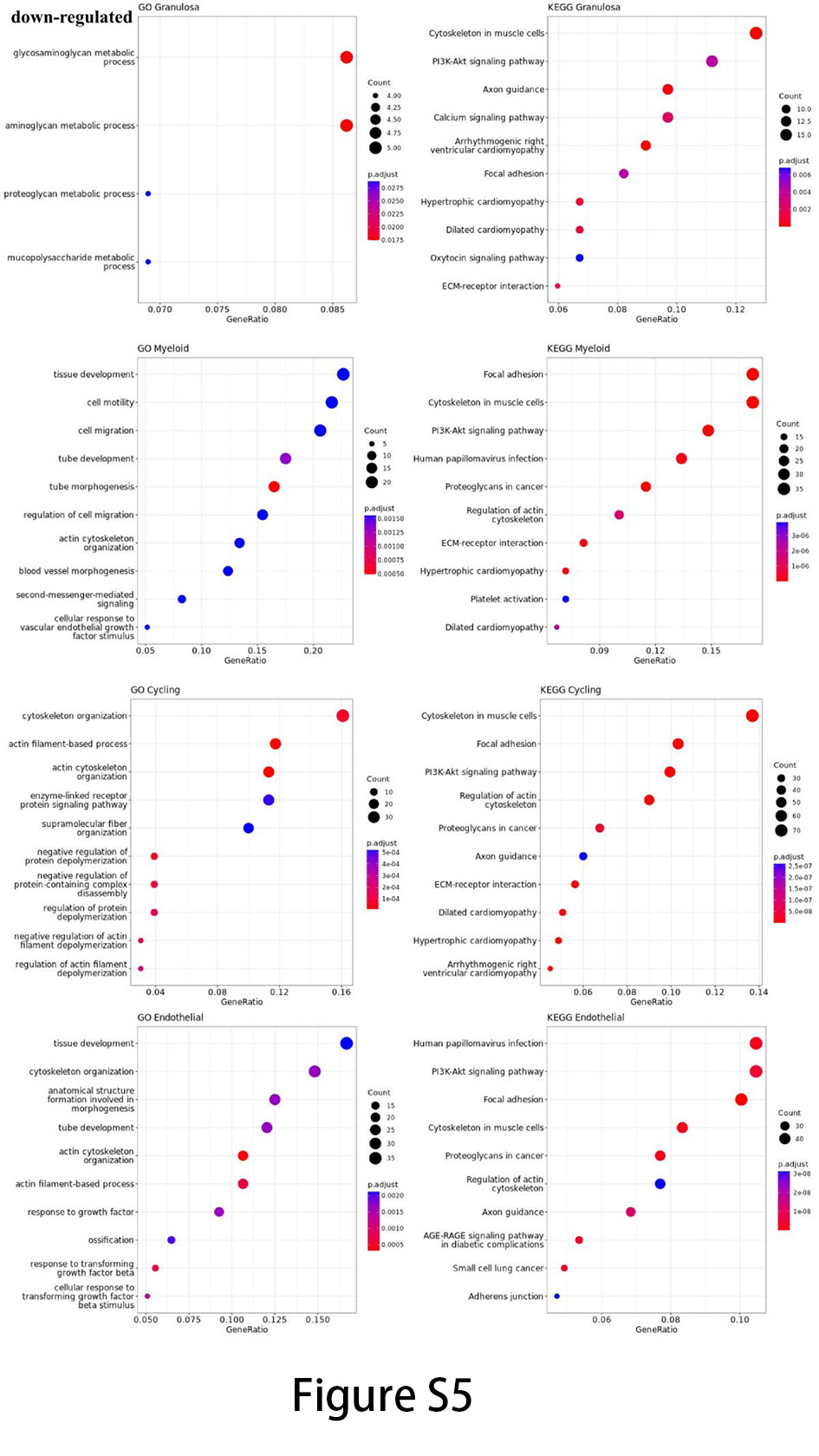

Supplement: Supplementary file 1 [file biology-14-01351-s001.zip › Figure S5.jpg]

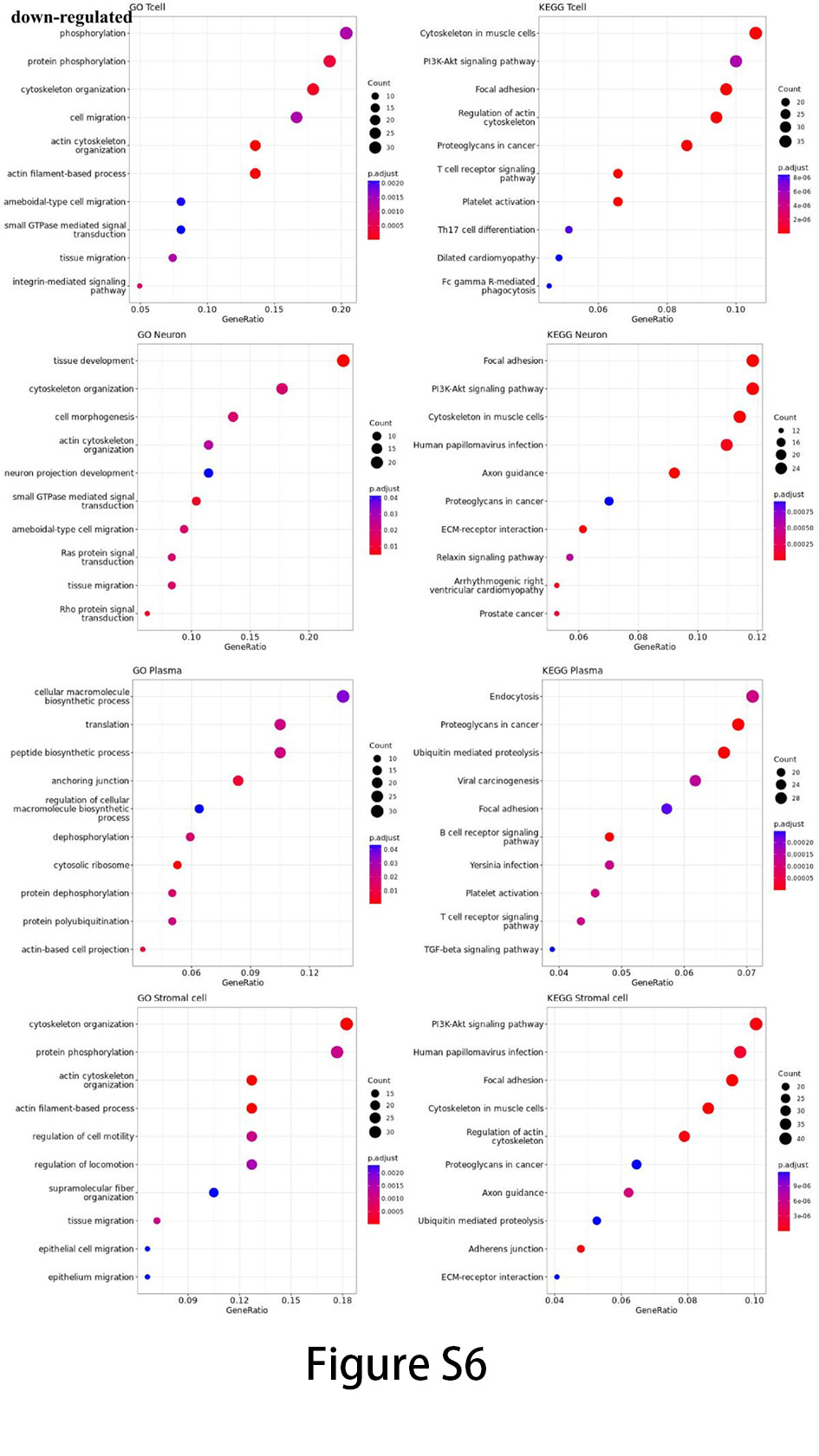

Supplement: Supplementary file 1 [file biology-14-01351-s001.zip › Figure S6.jpg]

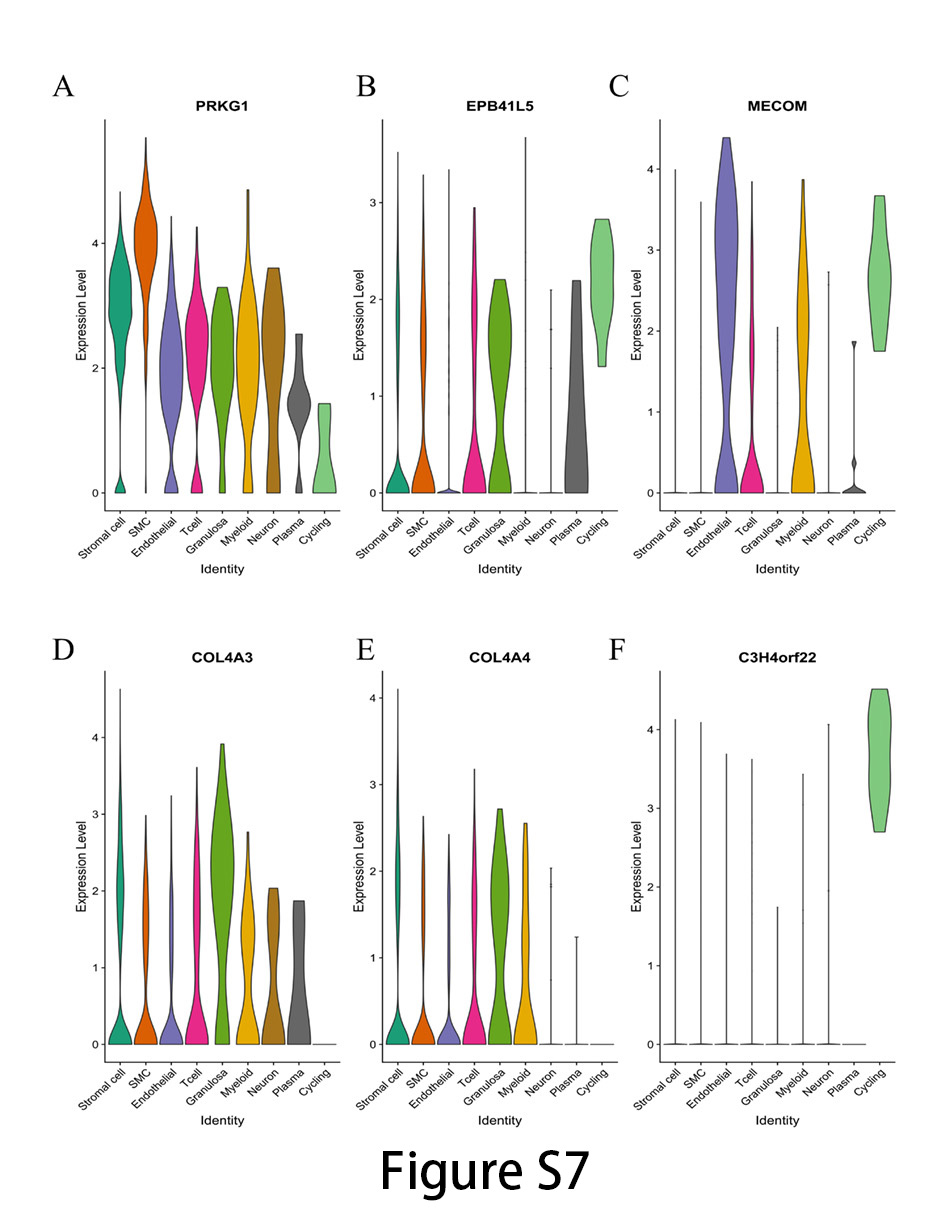

Supplement: Supplementary file 1 [file biology-14-01351-s001.zip › Figure S7.jpg]

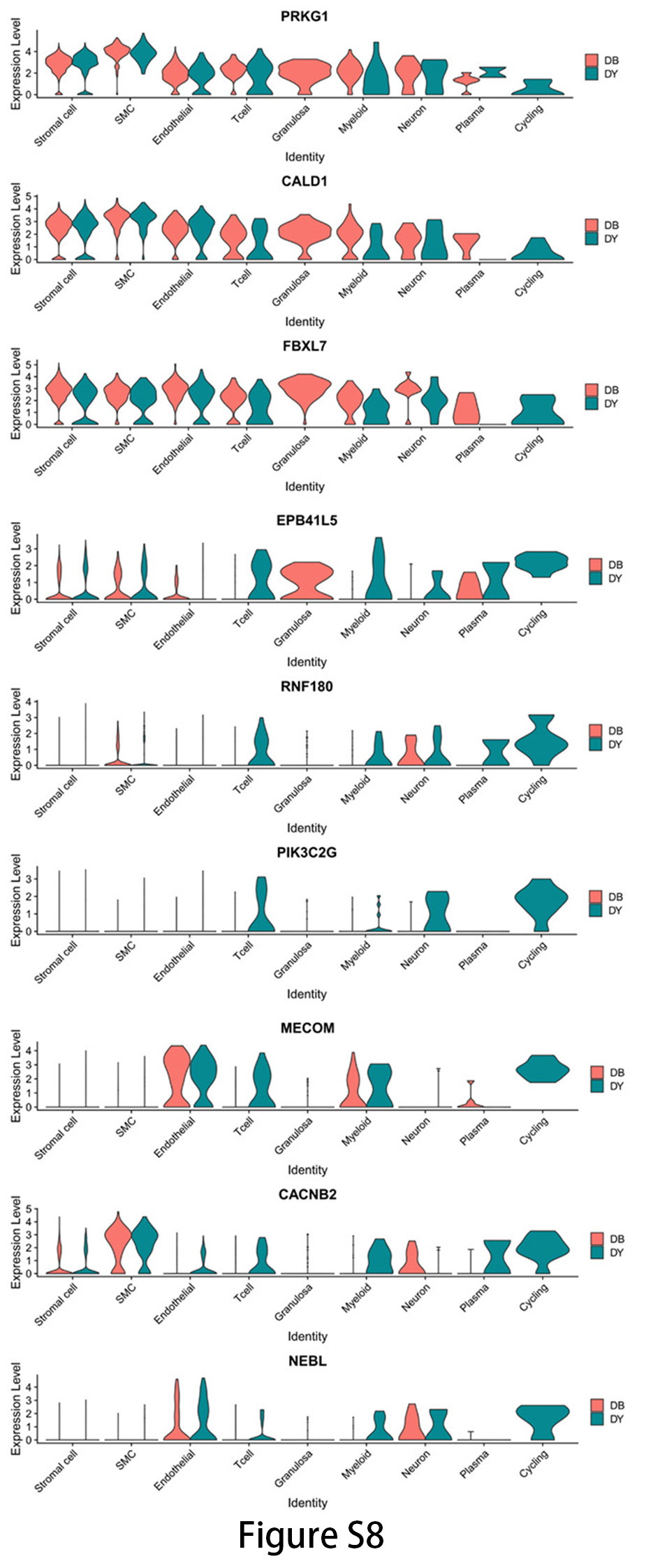

Supplement: Supplementary file 1 [file biology-14-01351-s001.zip › Figure S8.jpg]
